# Economic co-production of poly(malic acid) and pullulan from Jerusalem artichoke tuber by *Aureobasidium pullulans* HA-4D

**DOI:** 10.1186/s12896-017-0340-y

**Published:** 2017-02-23

**Authors:** Jun Xia, Jiaxing Xu, Xiaoyan Liu, Jiming Xu, Xingfeng Wang, Xiangqian Li

**Affiliations:** 1 Jiangsu Key Laboratory for Biomass-Based Energy and Enzyme Technology, Jiangsu Collaborative Innovation Center of Regional Modern Agriculture and Environmental Protection, College of Chemistry and Chemical Engineering, Huaiyin Normal University, Huai’an, 223300 China; 2 Jiangsu Province Engineering Laboratory for Biomass Conversion and Process Integration, Huaiyin Institute of Technology, Huai’an, 223300 China

**Keywords:** Poly(malic acid), Pullulan, Jerusalem artichoke, Co-production, Enzyme activity

## Abstract

**Background:**

poly(L-malic acid) (PMA) is a water-soluble polyester with many attractive properties in medicine and food industries, but the high cost of PMA fermentation has restricted its further application for large-scale production. To overcome this problem, PMA production from Jerusalem artichoke tubers was successfully performed. Additionally, a valuable exopolysaccharide, pullulan, was co-produced with PMA by *Aureobasidum pullulans* HA-4D.

**Results:**

The Jerusalem artichoke medium for PMA and pullulan co-production contained only 100 g/L hydrolysate sugar, 30 g/L CaCO_3_ and 1 g/L NaNO_3_. Compared with the glucose medium, the Jerusalem artichoke medium resulted in a higher PMA concentration (114.4 g/L) and a lower pullulan concentration (14.3 g/L) in a 5 L bioreactor. Meanwhile, the activity of pyruvate carboxylase and malate dehydrogenas was significantly increased, while the activity of α-phosphoglucose mutase, UDP-glucose pyrophosphorylase and glucosyltransferase was not affected. To assay the economic-feasibility, large-scale production in a 1 t fermentor was performed, yielding 117.5 g/L PMA and 15.2 g/L pullulan.

**Conclusions:**

In this study, an economical co-production system for PMA and pullulan from Jerusalem artichoke was developed. The medium for PMA and pullulan co-production was significantly simplified when Jerusalem artichoke tubers were used. With the simplified medium, PMA production was obviously stimulated, which would be associated with the improved activity of pyruvate carboxylase and malate dehydrogenas.

**Electronic supplementary material:**

The online version of this article (doi:10.1186/s12896-017-0340-y) contains supplementary material, which is available to authorized users.

## Background

Poly(*β*-malic acid) (PMA) is a water-soluble polyester consisting of malic acid monomer. Since PMA has many attractive properties, including biocompatibility, biodegradability and can be easily chemically modified, this polyester has attracted great interest for its potential in biomedical and food applications [1, 2]. For example, Ljubimova et al. have developed a new drug delivery system based on PMA and called it Polycefins™ [3]. Polycefins™ was of direct tumor targeting and showed a significant decrease in the tested cancer cell viability [4, 5]. Currently, Polycefins™ is at the step of preclinical trials and is considered to be a promising candidate for anti-cancer treatment. On the other hand, the only monomer of PMA, malic acid, is predominantly used in the food and beverage industries as an acidulant and taste modifier [6]. Several microorganisms including *Aspergillus* species, *Schizophyllum commune* and engineered *Escherichia coli* are capable of producing malic acid [7, 8]. Among these microbes, *A.flavus* can produce malic acid at the highest titer of 113 g/L [7]. However, *A.flavus* is a pathogenic microbe thus its industrial application has been prevented [9], and the production of malic acid by genetically engineered *E.coli* is severely limited by the end-product inhibition because of the strong acidity of malic acid [8]. By contrast, PMA is not toxic to cells, thus it can be accumulated to a high concentration in the culture broth. It was found that some strains of *Aureobasidium* spp. could produce large quantities of PMA under suitable conditions [2, 10], *Aureobasidium* spp. is nonpathogenic and thus it is suitable for industrial application. Therefore, microbial fermentation of PMA followed by acid hydrolysis provides a promising route for commercial malic acid production. For example, Zou et al. [2] investigated the feasibility of malic acid production derived from PMA. They found that *A.pullulans* ZX-10 could produce PMA as the major product at a high titer of 125.5 g/L, and pure malic acid was then obtained from acid hydrolysis of PMA with a high recovery rate of 84%, this study offered an alternative method for malic acid production in the future.

The cost of microbial fermentation of PMA is high when glucose is used as the carbon source, research into employing other renewable and inexpensive feedstock for PMA production has attracted more attention. Lignocellulosic materials from agricultural by-products (e.g., corn fiber and wheat straw) are considered as primary carbon sources for biorefinery engineering, but the pretreatment of these materials is energy-consuming, and due to the low concentration of sugar and the existence of toxic compounds (furfural, 5-hydroxymethyl furfural, etc.) in the hydrolysate of these materials, only a relatively low concentration of PMA (20 ~ 30 g/L) was obtained from these agricultural biomass [11, 12]. Sweet potato is rich in starch, which can be hydrolyzed to glucose and used by the microorganisms in the fermentation process. Zan et al. [13] performed PMA production from sweet potato hydrolysate, the maximum concentration of PMA reached 57.5 g/L when the cells were immobilized in an aerobic fibrous-bed bioreactor. However, sweet potato is a staple food crop in parts of Africa, Asia and the Pacific [14], the large-scale production of PMA from sweet potato may create competition with humans for food. To overcome the aforementioned problems, the utilization of a non-grain crop containing a high level of carbohydrates for PMA production is necessary.

Jerusalem artichoke (*Helianthus tuberosus* L.) is a low-cost and widely available non-grain crop, this species does not interfere with food crop production because it can grow well in barren lands, giving a yield of about 90 t/ha by fresh weight. Jerusalem artichoke tuber is rich in carbohydrates, of which 70–90% (w/w) is inulin [14], and inulin can be easily hydrolyzed to monomeric sugars (fructose and glucose) for microbial fermentation. Thus Jerusalem artichoke is a promising raw material for biorefinery engineering [15]. To date, Jerusalem artichoke has been employed for the production of several biochemical products, such as 2,3-butanediol, docosahexaenoic acid and succinic acid [16–18]. Therefore, Jerusalem artichoke may be a suitable feedstock instead of glucose for microbial fermentation of PMA.

In addition to the utilization of low-cost raw materials, enriching the diversity of the products is also useful to make the fermentation more economical. Recent research showed that some PMA-producing strains could produce pullulan as a by-product [19, 20]. Pullulan is a linear homopolysaccharide which is made mainly of maltotriose repeating units interconnected by *α-*1,6 linkages [21]. This polysaccharide is of economic importance and has been commercially produced by the Hayashibara Company (Japan) since 1959 [22]. Pullulan has been applied in extensive applications such as food, pharmaceutical and chemical industries. For example, pullulan can be used to make capsules because this polysaccharide has no mutagenic and toxicological activities. NPcaps® capsules, which are made of pullulan, have been successfully brought to commercial market and used for addressing pharmaceuticals and dietary supplements [23]. In our previous study, a high PMA-producing strain, *A.pullulan* HA-4D, was isolated and a high production of PMA was obtained with this strain [24]. We found that strain HA-4D could also produce an unknown exopolysaccharide as a by-product, which was probably pullulan according to the literature. In the present study, an economical co-production system for PMA and pullulan from Jerusalem artichoke was developed. PMA production was obviously stimulated with the use of Jerusalem artichoke tuber hydrolysate. The underlying mechanism was discussed with regard to the key enzymes activities involved in vital pathways of PMA and pullulan biosynthesis.

## Methods

### Microorganism and medium

*A.pullulans* HA-4D (CGMCC No.7.208), which can co-produce PMA and pullulan, was used in this study. *A.pullulans* HA-4D was maintained on potato dextrose agar slants. The composition of seed medium was 80 g/L glucose, 1 g/L Yeast extract, 2 g/L NaNO_3_, 0.1 g/L KH_2_PO_4_, 0.1 g/L MgSO_4_, 0.5 g/L KCl and 20 g/L CaCO_3_. The standard PMA production medium (GM medium) consisted of 100 g/L glucose, 2 g/L NaNO_3_, 0.1 g/L KH_2_PO_4_, 0.1 g/L MgSO_4_, 0.5 g/L KCl, 0.1 g/L ZnSO_4_ and 30 g/L CaCO_3_. The optimized fermentation medium (JAT medium) contained 100 g/L hydrolysate sugar (hydrolysate of Jerusalem artichoke tuber), 1 g/L NaNO_3_ and 30 g/L CaCO_3_.

### Preparation of the Jerusalem artichoke tuber hydrolysate

Jerusalem artichoke tubers were harvested from farms in Xuzhou, Jiangsu Province, China in December, 2015. The washed tubers were peeled, sliced and dried. The dried tubers were ground into fine powder using a crusher. Acid hydrolysis was conducted by weighing 100 g of fine powder into 900 mL of 5% (v/v) H_2_SO_4_. The mixture was heated at 80 °C for 40 min. After hydrolysis, the hydrolysate was filtered with filter paper. The pH of the filtrate was adjusted to 6.5 with 1 M NaOH.

### Optimization of the medium for PMA and pullulan co-production

The medium optimization was performed in the shake-flask scale. Shake flask fermentation was carried out at 25 °C and 200 rpm for 7 days. Different concentrations of nutrient elements, including NaNO_3_, KH_2_PO_4_, MgSO_4_, KCl, ZnSO_4_ and CaCO_3_, were added to the medium for PMA and pulluan co-production, the nutrient elements that affect PMA and pullulan co-production by *A.pullulans* HA-4D were standardized by varying one factor at a time. The defined parameter of one experiment was followed for the succeeding experiments.

### Fed-batch fermentation for PMA and pullulan co-production

*A.pullulans* HA-4D was maintained on a potato dextrose agar slant and then transferred into a 500 mL flask containing 100 mL of the seed medium. The seed culture was aerobically incubated at 25 °C and 200 rpm for 48 h, then 300 mL of the seed culture was inoculated into 2.7 L of the fermentation medium in a 5 L stirred tank fermentor (Winpact, USA). Fermentation was performed at 25 °C under aeration at 1 vvm with a stirring speed of 400 rpm. The pH of the culture broth was maintained at approximately 6.5 with the addition of CaCO_3_. For fed-batch fermentation from JAT medium, when the concentration of reducing sugars in the culture broth decreased to 10 g/L, the concentrated Jerusalem artichoke tuber hydrolysate (approximately 300 g/L reducing sugars) was fed to maintain the reducing sugar at approximately 10 g/L. The feeding rate was in the range of 9 ~ 15 mL/h, and approximately 0.9 L of the feeding solution was added into the 5 L fermentor. The final volume of culture broth was about 3.5 L because of the losses from sampling and water evaporation. As a control, fed-batch fermentation from glucose was also carried out, the culture parameters were identical to those of JAT medium, except that the fermentation medium was GM medium and the feeding solution was composed of 300 g/L glucose.

Fermentation in a 1 t fermentor was carried out in Baimai Co., Ltd. (Huai’an, China). For the seed culture, five slant eggplant flasks of *A.pullulans* HA-4D were inoculated into a 100 L fermentor containing 60 L of the seed medium. The seed culture was incubated at 25 °C for 48 h. Then the seed culture was transferred into a 1 t fermentor containing 0.54 t of the JAT medium. The aeration was 1 vvm and the stirring speed was 250 rpm. The feeding rate was in the range of 1.8 ~ 2.3 L/h to maintain the residual sugar at approximately 10 g/L. The other parameters were identical to those for fed-batch fermentation in a 5 L fermentor.

### Purification and characterization of pullulan

The culture broth was centrifuged and the resulting supernatant was used for purification of exopolysaccharide and PMA. These two biopolymers were purified by the repeated ethanol precipitation [19]. The first addition of 1 volume of cold ethanol to the supernatant was to selectively purify exopolysaccharide as precipitates, the precipitated exopolysaccharide was then subjected to dialysis for structure characteristics. After the removal of the exopolysaccharide, the supernatant was added with 2 volume of ethanol, the mixture was kept at 4 °C for overnight for precipitation of PMA.

The purified exopolysaccharide was characterized by Fourier transform infrared spectroscopy (FT-IR), ^1^H and ^13^C nuclear magnetic resonance (NMR) spectra. FT-IR was performed on a Nicolet 560 FT-IR spectrometer (Thermo, USA) and the sample was blended with KBr pellet. The ^1^H and ^13^C-NMR spectra were recorded using a Bruker AVANCE AV-400 spectrophotometer (Bruker Biospin Corp., Billerica, MA), 10 mg of the purified exopolysaccharide was dissolved in 0.5 mL DMSO-d_6_ solvent and TMS was used as an internal standard for proton NMR [25].

### Assay of enzyme activity

Cells grown in fed-batch fermentation in 5 L fermentor at early stage (40 h) and later stage (100 h) were collected for the measurement of intracellular enzyme activity. All procedures were carried out at 4 °C. The collected cells were washed twice with 0.85% NaCl solution. The harvested cells were suspended in 0.2 M phosphate buffer (pH 7.0). The suspended cells were disrupted by sonication and then centrifuged at 12,000 × *g* at 4 °C for 10 min. The cell debris was removed by centrifugation. The supernatant was collected as the cell-free extract and used for enzyme activity determination. Reactions were initiated by adding the cell-free extract to yield a final volume of 1 mL. The pyruvate carboxylase (PYC; EC 6.4.1.1), malate dehydrogenase (MDH; EC 1.1.1.37) and glucosyltransferase (FKS; EC 2.4.1.34) activity were assayed according to previous reports [26–28]. The α-phosphoglucose mutase (PGM; EC 5.4.2.2) and UDP-glucose pyrophosphorylase (UDPG; EC 2.7.7.9) activity were determined with a commercial ELISA Kit (Haochen-Bio, Shanghai, China) according to the manufacturer’s instructions. The total protein concentration in the crude cell extract was measured by the Bradford method, with bovine serum albumin as a standard.

### Analytical methods

The concentrations of glucose and fructose in the Jerusalem artichoke tuber hydrolysate were analyzed by HPLC (Agilent 1260 series, USA), with an Aminex HPX-87P column (300 × 7.8 mm; Bio-Rad, USA) and a refractive index detector. HPLC analysis was performed with deionized water as the mobile phase and a flow rate of 0.5 mL/min at 80 °C. The total sugar of the hydrolysate and the concentration of residual sugar during fed-batch fermentation was determined using the DNS method [29]. The total nitrogen content of Jerusalem artichoke tuber hydrolysate was determined by the Kjeldahl method with a Kjeldahl apparatus SKD-800 (Peiou, Shanghai, China) following the manufacturer’s instructions.

The concentration of PMA was estimated from the concentration of malic acid after hydrolysis. The fermentation broth was centrifuged and the resulting supernatant was mixed with an equal volume of 2 M H_2_SO_4_, the mixture was incubated at 90 °C for 9 h. After neutralization of the solution, the malic acid concentration was determined by HPLC as described previously [24]. For pullulan analysis, 1 volume of ethanol was added to the supernatant and incubated at 4 °C overnight. The precipitates were centrifuged at 8000 × *g* for 10 min and dried at 80 °C to a constant weigh [19].

## Results

### Hydrolysis of Jerusalem artichoke tubers

The major component of Jerusalem artichoke tubers is inulin, which is composed of multiple fructose units terminated by a glucose unit. *A.pullulans* has a relatively low inulinase activity (0.05 U/mL), such a low activity was unsatisfactory for exopolysaccharide or PMA production [30]. Therefore, the inulin in the medium must be subjected to hydrolysis prior to fermentation. Given its strong acid strength for cleaving inulin bonds, sulphuric acid was employed for the saccharification of Jerusalem artichoke tubers. As shown in Table 1, the Jerusalem artichoke tubers used in this study contained 20.4 ± 0.5 g/L dry weight. After the optimization of hydrolysis parameters (data not shown), 78.4 ± 0.6 g/L reducing sugar was released to the hydrolysate, which contained 58.2 ± 0.4 g/L fructose and 12.7 ± 0.6 g/L glucose. In addition to reducing sugars, the hydrolysate contained 0.68 ± 0.03 g/L nitrogen and several kinds of mineral elements, such as potassium, phosphorus, magnesium and calcium [31].

**Table 1:** Nutritional composition of Jerusalem artichoke tuber

Composition	Content	Reference
Dry weight (g/100 g wet weight)	20.4 ± 0.5	This study
Total nitrogen (g/100 g dry weight)	0.68 ± 0.03	This study
Fructose (g/100 g dry weight hydrolysate)	58.2 ± 0.4	This study
Glucose (g/100 g dry weight hydrolysate)	12.7 ± 0.6	This study
Total sugar (g/100 g dry weight)	78.4 ± 0.6	This study
Potassium (mg/100 g dry weight)	2335	[31]
Phosphorus (mg/100 g dry weight)	389	[31]
Magnesium (mg/100 g dry weight)	189	[31]
Calcium (mg/100 g of dry weight)	186	[31]
Sulfur (mg/100 g of dry weight)	62	[31]

### Optimization of the JAT medium for PMA and pullulan co-production

**Fig. 1:**
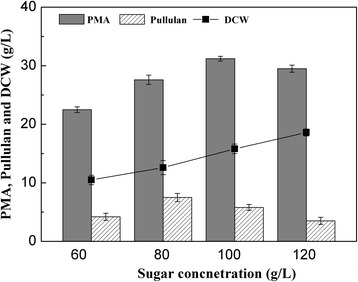
Effect of initial sugar concentration derived from Jerusalem artichoke tuber hydrolysate on PMA and pullulan co-production in shake-flasks

To determine the optimal sugar concentration derived from the Jerusalem artichoke tuber hydrolysate on PMA production, the initial sugar concentrations from 60 to 120 g/L were tested. As shown in Fig. 1, the increased sugar concentration was favorable for cell growth within the range of 60 to 120 g/L, the highest dry cell weight (DCW) concentration of 18.6 ± 0.6 g/L was achieved when the sugar concentration was 120 g/L, which was probably attributed to the high nitrogen content in the Jerusalem artichoke tuber hydrolysate. PMA production was improved when the initial sugar concentration increased from 60 g/L to 100 g/L. The optimal concentration of initial sugar concentration for PMA production was 100 g/L and PMA concentration reached the highest value (31.2 ± 0.4 g/L), further increasing the sugar concentration depressed PMA biosynthesis. Similar results were also observed on pullulan production by *A.pullulans* HA-4D, the optimal sugar concentration for pullulan production was 80 g/L. To obtain the highest yield of PMA, the initial sugar concentration of 100 g/L derived from the Jerusalem artichoke tuber hydrolysate was used for the subsequent experiments.

In addition to monomeric sugar, the Jerusalem artichoke tuber hydrolysate also contains some mineral nutrients and vitamins, which might satisfy the nutrient demand of microorganisms for cell growth and product yield. Therefore, the standard PMA-producing medium might be simplified with the use of Jerusalem artichoke tuber hydrolysate. As shown in Table 2, several concentrations of the required nutrients, such as NaNO_3_, KH_2_PO_4_ and CaCO_3_ were tested. The results showed that except for NaNO_3_ (optimal concentration of 1 g/L, *P* < 0.05) and CaCO_3_ (optimal concentration of 30 g/L, *P* < 0.01), no significant enhancements on PMA production were achieved when mineral nutrients were added to the medium containing the Jerusalem artichoke tuber hydrolysate. Meanwhile, pullulan yield was significantly decreased with the addition of CaCO_3_ (*P* < 0.01) and cell growth was enhanced with the addition of NaNO_3_ (*P* < 0.05). Therefore, when Jerusalem artichoke tuber hydrolysate was employed as the carbon source for PMA production, no extra components, except for NaNO_3_ and CaCO_3_, were added to the medium, which would further decrease the cost of PMA fermentation.

**Table 2:** Effects of different supplements on the co-production of PMA and pullulan by *A.pullulans* HA-4D in JAT fermentation medium. Shake flask fermentation was carried out at 25 °C and 200 rpm for 7 days

Components	Concentration (g/L)	PMA (g/L)	Pullulan (g/L)	DCW (g/L)
NaNO_3_	0	31.9 ± 0.5	5.3 ± 0.4	12.5 ± 0.3
	1	33.2 ± 0.4*	6.3 ± 0.4	13.9 ± 0.4*
	2	30.8 ± 0.4	5.8 ± 0.4	15.8 ± 0.5**
KH_2_PO_4_	0	34.0 ± 0.4	6.1 ± 0.5	12.8 ± 0.4
	0.1	33.4 ± 0.4	6.3 ± 0.4	13.9 ± 0.9
	0.2	32.8 ± 1.1	6.6 ± 0.5	14.3 ± 0.8
MgSO_4_	0	35.1 ± 0.3	5.8 ± 0.5	13.2 ± 0.5
	0.1	34.1 ± 0.8	6.1 ± 0.3	12.9 ± 0.6
	0.2	33.7 ± 0.9	6.7 ± 0.3	12.5 ± 0.4
KCl	0	36.3 ± 0.5	5.5 ± 0.4	11.9 ± 0.2
	0.5	35.1 ± 0.5	5.7 ± 0.4	12.5 ± 0.4
	1	34.6 ± 0.9	6.4 ± 0.3	12.9 ± 0.8
ZnSO_4_	0	36.8 ± 0.4	6.0 ± 0.3	12.5 ± 0.4
	0.1	36.2 ± 0.6	5.5 ± 0.4	11.9 ± 0.4
	0.2	35.8 ± 0.4	5.1 ± 0.5	11.6 ± 0.3
CaCO_3_	0	26.3 ± 0.4	9.7 ± 0.6	11.5 ± 0.4
	15	33.9 ± 0.6**	6.8 ± 0.2**	11.9 ± 0.5
	30	36.6 ± 0.7**	6.0 ± 0.4**	12.4 ± 0.9

*0.01 < *P* < 0.05

**P < 0.01

### Analysis of the co-fermentation products

**Fig. 2:**
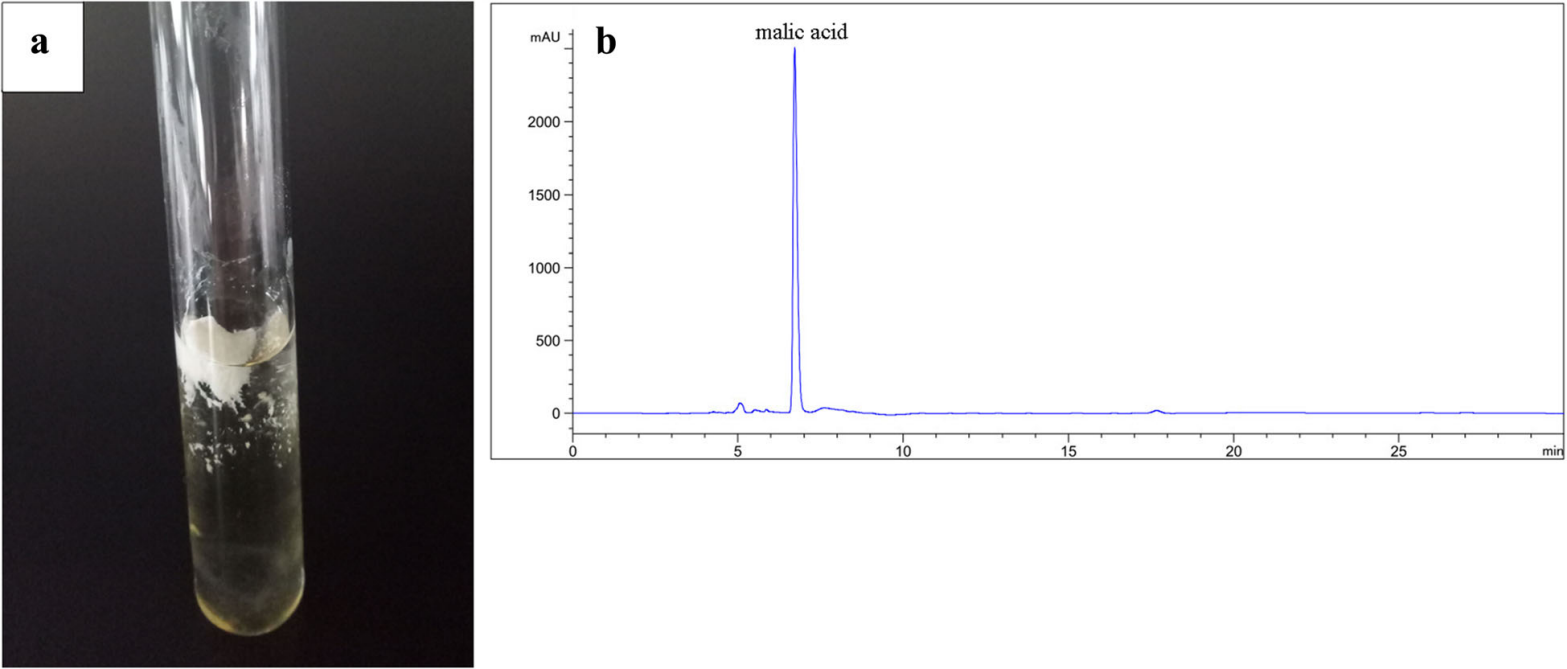
Precipitation of exopolysaccharide with the addition of ethanol (**a**) and HPLC analysis of the supernatant after the removal of exopolysaccharide (**b**)

In addition to PMA, we found that *A.pullulans* HA-4D also produced an unknown exopolysaccharide as a by-product. As shown in Fig. 2a, the exopolysaccharide was pigment-free and precipitated on the upper layer of the culture broth. *A.pullulans* is a biotechnologically important fungus due to its high yield of the commercial polysaccharide, pullulan. To verify if the exopolysaccharide produced by *A.pullulans* HA-4D is also pullulan, the purified exopolysaccharide was subjected to structural characterization using FT-IR and NMR spectra. The absorption band in the FT-IR spectrum (Additional file 1: Figure S1a) at 3394 cm^−1^ and 2928 cm^−1^ were characteristic of O-H stretching and C-H stretching, respectively. The characteristic signals appeared at 1637, 1420, 1152 and 1020 cm^−1^ were due to O-C-O stretching, C-O-H bending, C-O-C stretching and C-O stretching, respectively [25]. Furthermore, the signals arrived in the ^1^H-NMR spectrum (Additional file 1: Figure S1b) between 4 and 5.5 ppm infers proton carrying carbon atoms attached to an electronegative atom, signals at 1.0 ppm was due to 6-deoxy-D-altrose present in the pullulan [25]. ^13^C-NMR for the purified exopolysaccharide was shown in Additional file 1: Figure S1c, the anomeric carbon region shows three signals corresponding to *α*-(1 → 4) (101.8, 101.1 ppm) and *α*-(1 → 6) (99.2 ppm) linkages, splitting of C-6 (60.7, 61.3 ppm) and C-4 (80.4, 81.2 ppm) were due to C-1 of (1 → 4) linked glucose unit, the signal resonance at 67.4 ppm corresponds to C-6 of the 1,6-linked *α*-D-glucose [32]. Based on the above results, the exopolysaccharide produced by *A.pullulans* HA-4D was confirmed to be pullulan.

After the removal of pullulan, the fermentation broth was subjected to acid hydrolysis and PMA was hydrolyzed to malic acid. As shown in Fig. 2b, the dominant component of the fermentation broth hydrolysate was malic acid, only a few impurities existed in the fermentation broth. The kinetics of the acid hydrolysis of PMA had been investigated previously, a very high recovery rate (84 ~ 99%) of malic acid from PMA was easily obtained by a simple acid hydrolysis method [2, 10]. *A.pullulans* HA-4D could produce a high amount of PMA under suitable conditions [24], thus we think that *A.pullulans* HA-4D can be a good candidate for high malic acid production with high purity, and the co-produced pullulan could enrich the diversity of the products and improve the economical-feasibility of the fermentation process.

### Fed-batch fermentation for the co-production of PMA and pullulan

**Fig. 3:**
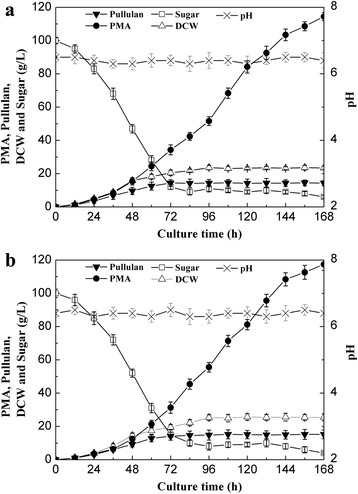
PMA and pullulan co-production with JAT medium in fed-batch fermentation by *A.pulluans* HA-4D. **a** 5 L fermentor; (**b**) 1 t fermentor

To scale up PMA and pullulan co-production from Jerusalem artichoke tubers, 5 L fermentation was carried out with the JAT medium. Our previous study demonstrated that fed-batch fermentation favored the high-level accumulation of PMA by *A.pullulans* HA-4D [24], thus fed-batch fermentation with a continuous feeding strategy was carried out in this study. The time course of the PMA, pullulan, biomass and residual sugar concentration was shown in Fig. 3a. After 168 h of fermentation, the final PMA, pullulan and biomass concentration achieved 114.4 ± 2.4 g/L, 14.3 ± 1.5 g/L and 23.4 ± 1.1 g/L, respectively. The PMA and pullulan yield on sugar was 0.74 g/g and 0.09 g/g, respectively. As a control, PMA and pullulan co-production using GM medium was also carried out (data not shown), the resulting concentrations of PMA, pullulan and biomass reached 96.2 ± 1.8 g/L, 20.8 ± 1.3 g/L and 20.2 ± 0.8 g/L, respectively. Meanwhile, PMA and pullulan yield on sugar with GM medium was 0.62 g/g and 0.14 g/g, respectively. The concentration of PMA and pullulan obtained with JAT medium was 18.9% higher and 31.3% lower, respectively, compared with those with GM medium, this result indicated that JAT medium was beneficial for PMA biosynthesis but unfavorable for pullulan production. On the other hand, the better cell growth obtained from JAT medium might be attributed to the abundant nitrogen content of the Jerusalem artichoke tubers.

Furthermore, to examine the feasibility of large-scale PMA and pulluan co-production with JAT medium, fed-batch fermentation was scaled up to a 1 t fermentor. As shown in Fig. 3b, the pullulan and PMA production was comparable to that of a 5 L fermentor. The final PMA and pullulan concentration reached 117.5 ± 3.7 g/L and 15.2 ± 2.6 g/L, respectively, thereby suggesting that PMA and pullulan co-production from Jerusalem artichoke tuber is a promising route for industrial purpose. The production levels of PMA and pullulan by different strains were summarized in Table 3 [13, 19, 20, 33–36]. Compared with the co-production of PMA and pullulan by other strains, the PMA and pullulan levels in this work were superior to other reports in literature, but inferior to the highest level of the individual production of PMA or pullulan. Therefore, the co-production system for PMA and pullulan in this study was practical and economical. Through this co-production system, the cost of raw materials was significantly decreased, and a valuable exopolysaccharide was co-produced to improve the economics of the system.

**Table 3:** Comparison of PMA and pullulan production by different strains in different fermentation processes

Strain	Substrate	PMA (g/L)	Pullulan (g/L)	Time (h)	Reference
*A.pullulans* RSU7	glucose	8.9	9.8	168	[20]
*A.pullulans* CCTCC M2012223	glucose	46.5	28.8	60	[19]
	HSP	57.5	ND	156	[13]
*A.pullulans* var. *pullulans* MCW	glucose	152.5	ND	120	[33]
*A.pullulans* ipe-1	glucose	60	ND	72	[34]
*A.pullulans* Y68	glucose	ND	59	60	[35]
*A.pullulans* var. *melanogenium* P16	sucrose	ND	67.4	132	[36]
*A.pullulans* HA-4D	glucose	96.2	20.8	168	This study
	HJAT	117.5	15.2	168	

*HSP* Hydrolysate of sweet potato, *HJAT* Hydrolysate of Jerusalem artichoke tuber, *ND* No data

### Enzyme activity in the biosynthesis of PMA and pullulan

**Fig. 4:**
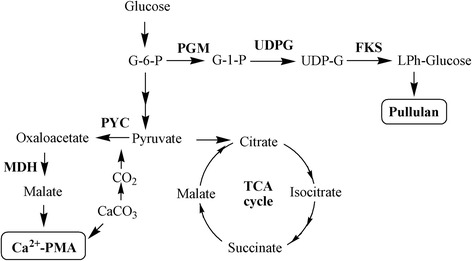
The proposed pathway of PMA and pullulan metabolism in *A.pullulans*

Pullulan and PMA can be produced simultaneously due to their tightly interrelated metabolic pathway, the proposed metabolism pathways of PMA and pullulan are shown in Fig. 4. Pyruvate carboxylase (PYC; EC 6.4.1.1) and malate dehydrogenase (MDH; EC 1.1.1.37) are the key enzymes in the biosynthetic pathway of malic acid, α-phosphoglucose mutase (PGM; EC 5.4.2.2), UDP-glucose pyrophosphorylase (UDPG; EC 2.7.7.9) and glucosyltransferase (FKS; EC 2.4.1.34) are generally regarded as key enzymes involved in pullulan synthesis [37]. To demonstrate the effect of the Jerusalem artichoke tuber hydrolysate on the intracellular carbon flux, the key enzymes involved in the biosynthetic pathway of PMA and pullulan were assayed. Cells at early stage (40 h) and later stage (100 h) of fermentation process were collected to analyze the enzyme activity. As shown in Table 4, the PYC activity was significantly enhanced at both phases (40 h: *P* < 0.01; 100 h: *P* < 0.05) when JAT medium was used. Higher MDH activity was also observed when *A.pullulans* HA-4D was grown on JAT medium than on GM medium. By contrast, the activity of PGM, UDPG and FKS was not significantly affected. PYC and MDH are the key enzymes of malic acid biosynthesis pathway, thus the improved activities of PYC and MDH were beneficial for malic acid production. Jerusalem artichoke tuber contains several vitamins, including vitamin A, B_1_, B_2_, B_3_, B_6_, B_7_ and C [38]. Biotin (vitamin B_7_) is the key cofactor for PYC, the sufficient biotin available in the Jerusalem artichoke tuber may stimulate PYC activity so that PMA production was enhanced. Similar results were reported by other researchers. For example, Zou et al. [39] investigated the effects of cofactor on PMA production, and they found that PMA concentration was increased from 14.3 g/L to 23.1 g/L with the addition of 70 mg/L biotin. Therefore, the improved PYC and MDH activity might cause the global metabolic flux redistributions, thus the reductive pathway of malic acid would be strengthened by the use of JAT medium. Consequently, PMA production was enhanced.

**Table 4:** Activity of key enzymes involved in PMA and pullulan biosynthesis when *A.pullulans HA-4D* was grown in JAT medium or GM medium. PYC: pyruvate carboxylase; MDH: malate dehydrogenase; PGM: α-phosphoglucose mutase; UDPG: UDP-glucose pyrophosphorylase; FKS: glucosyltransferase

Time	Medium	Specific enzyme activities (U/mg protein)				
		PYC	MDH	PGM	UDPG	FKS
40 h	GM	0.18 ± 0.03	4.55 ± 0.44	(7.86 ± 0.40) × 10^−3^	(56.89 ± 5.88) × 10^−3^	0.79 ± 0.05
	JAT	0.31 ± 0.02**	5.37 ± 0.30	(8.25 ± 0.19) × 10^−3^	(53.41 ± 3.95) × 10^−3^	0.85 ± 0.03
100 h	GM	0.10 ± 0.02	2.52 ± 0.43	(6.29 ± 0.37) × 10^−3^	(25.51 ± 3.35) × 10^−3^	0.68 ± 0.04
	JAT	0.19 ± 0.03*	2.88 ± 0.22	(5.86 ± 0.42) × 10^−3^	(27.86 ± 4.09) × 10^−3^	0.65 ± 0.05

*0.01 < *P* < 0.05

**P < 0.01

## Discussion

Jerusalem artichoke tuber is rich in carbohydrates, of which 70–90% (w/w) is inulin [14], and inulin can be easily hydrolyzed to monomeric sugars (fructose and glucose) for microbial fermentation. Enzymatic and acidic hydrolysis are the most commonly used pretreatment methods for Jerusalem artichoke tuber. Commercial inulinase hydrolysis is usually carried out at 55 °C for 12 h, with an enzyme loading of 2.0 U/g substrate [17]. To reduce the cost of purchasing commercial inulinase, the mixed culture of two different strains is often employed. For example, Shin et al. [30] performed pullulan production from Jerusalem artichoke using a mixed culture of *A.pullulans* and *Kluyveromyces fragilis. K.fragilis* was introduced as an inulinase producer to accelerate the hydrolysis of inulin into fructose, and *A.pullulans* served as pullulan producer, the maximum pullulan production reached 15.5 g/L with this mixed culture system. In addition to enzymatic hydrolysis, acid hydrolysis is also often employed for the pretreatment of Jerusalem artichoke tuber. Yu et al. [18] found that among sulphuric, nitric and hydrochloric acids, sulphuric acid was the optimal acid for the hydrolysis of Jerusalem artichoke tubers. Acid hydrolysis is usually carried out with 0.2 ~ 5% H_2_SO_4_ at a liquid-to-solid ratio of 10:1 at 100 °C for 1 h. In the present study, the yield of reducing sugars was 78.4 g sugar/100 g dry Jerusalem artichoke tuber, thus sulphuric acid hydrolysis is a better route compared to the costly and lengthy enzymatic hydrolysis for the saccharification of Jerusalem artichoke tubers.

Aside from fructose and glucose as the major components, the hydrolysate of Jerusalem artichoke tuber also contain some other nutrients including protein, mineral contents and vitamins [15]. Therefore, the medium for PMA and pullulan co-production was significantly simplified with the use of Jerusalem artichoke tuber hydrolysate, which is a significant advantage in terms of decreasing the cost and increasing the efficiency of PMA and pulluan co-production. Similar results were reported for the fermentation of other bioproducts. For example, Gunnarsson et al. [16] found that when Jerusalem artichoke tuber hydrolysate was employed for succinic acid production, the addition of yeast extract and other nutrients was not strictly required, and 49.7 g/L succinic acid was produced from the Jerusalem artichoke tuber hydrolysate without additional media supplementation. Gao et al. [40] conducted single-cell protein fermentation by a marine yeast *Cryptococcus aureus* G7a, 53.0 g of crude protein per 100 g of cell dry weight was obtained from the medium consisting of Jerusalem artichoke extract only supplemented with hydrolysate of soybean meal.

In addition to the hydrolysate of Jerusalem artichoke tuber, the addition of CaCO_3_ to the medium was indispensible for the biosynthesis of PMA. First, the presence of CaCO_3_ can maintain the pH between 6.0 and 6.5, which is quite important for PMA production because a low pH may result in the degradation of PMA (pH < 5.0) [41]. Besides, a low pH may also induce the formation of by-product (e.g., extracellular polysaccharides) and chlamydospore by some strains of *Aureobasidium* spp. [42], which is undesirable in PMA production. Another reason for this is the significant role of CaCO_3_ in the biosynthesis of malic acid. Previous studies demonstrated that malic acid was synthesized either via the carboxylation of pyruvate and subsequent reduction of oxaloacetate (the reductive pathway) or the tricarboxylic acid cycle (the oxidative pathway) [43]. Malic acid synthesis via the reductive pathway provides a maximal theoretical yield of 2 mol malic acid per mol of glucose [8]. The presence of CaCO_3_ induced the switch of malic acid generation to the reductive pathway [43], thereby providing massive amounts of malic acid as the precursor for PMA biosynthesis.

## Conclusions

In this study, an economical co-production system for PMA and pullulan from Jerusalem artichoke was developed. The medium for PMA and pullulan co-production was significantly simplified when Jerusalem artichoke tubers were used. With the simplified medium, the production of PMA and pullulan in 1 t fermentor reached 117.5 g/L and 15.2 g/L, respectively. Meanwhile, PMA production was obviously stimulated, which would be associated with the improved activity of pyruvate carboxylase and malate dehydrogenas. This study demonstrated the great potential of Jerusalem artichoke for the economical co-production of PMA and pullulan at industrial scale.

## Additional files

Additional file 1: Figure S1. Structural characterization of the exopolysaccharide with FT-IR spectrum (a), ^1^H-NMR spectrum (b) and ^13^C-NMR spectrum (c). (DOCX 275 kb)
Additional file 2: Raw data used for the production of Additional file 1: Figure S1a, Table 2 and Table 4. (XLSX 186 kb)

## Abbreviations

DCW: Dry cell weight
FKS: Glucosyltransferase
FT-IR: Fourier transform infrared spectroscopy
G-1-P: Glucose-1-phosphate
G-6-P: Glucose-6-phosphate
GM medium: The standard PMA production medium
JAT medium: The optimized fermentation medium
LPh: Phospholipds intermediate
MDH: Malate dehydrogenase
NMR: Nuclear magnetic resonance spectra
PGM: α-phosphoglucose mutase
PMA: Poly(L-malic acid)
PYC: Pyruvate carboxylase
UDPG: UDP-glucose pyrophosphorylase
UDP-G: Uridine 5’-diphosphate glucose
