# Supplementary figures and images for: Economic co-production of poly(malic acid) and pullulan from Jerusalem artichoke tuber by Aureobasidium pullulans HA-4D
Source: BMC Biotechnol. 2017 Feb 23;17:20. doi: 10.1186/s12896-017-0340-y (PMC5324199; doi:10.1186/s12896-017-0340-y)

**Fig.S1a**





**Fig.S1b**


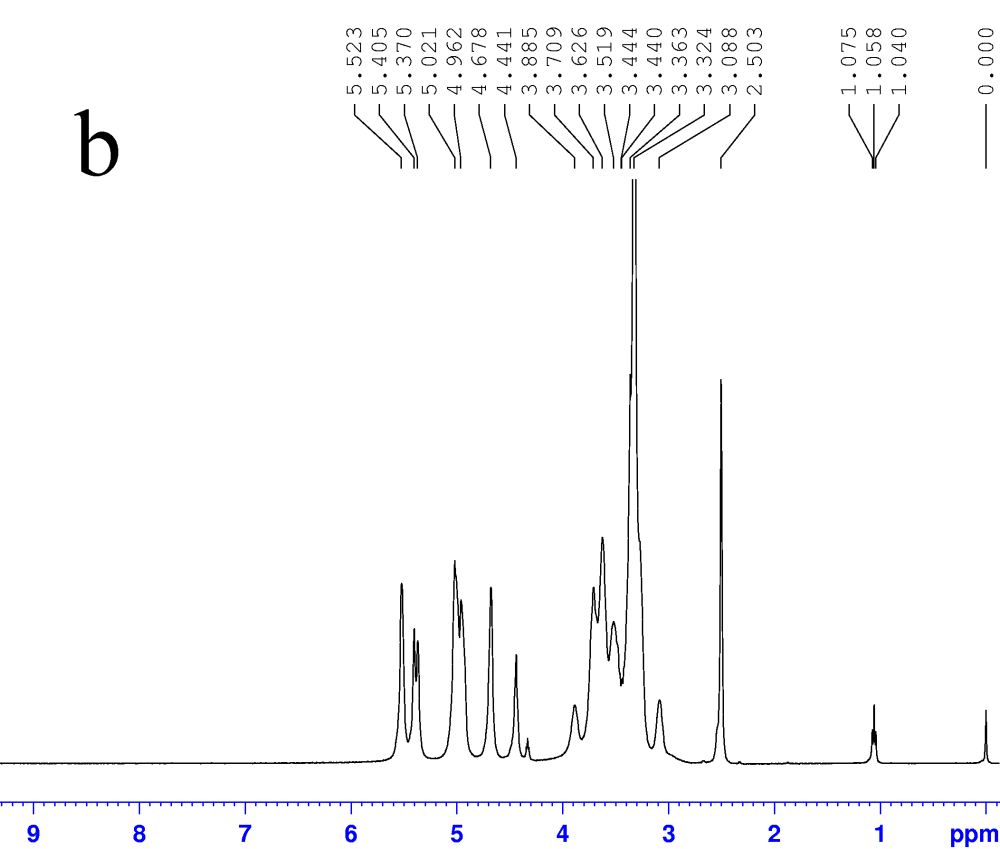


**Fig.S1c**


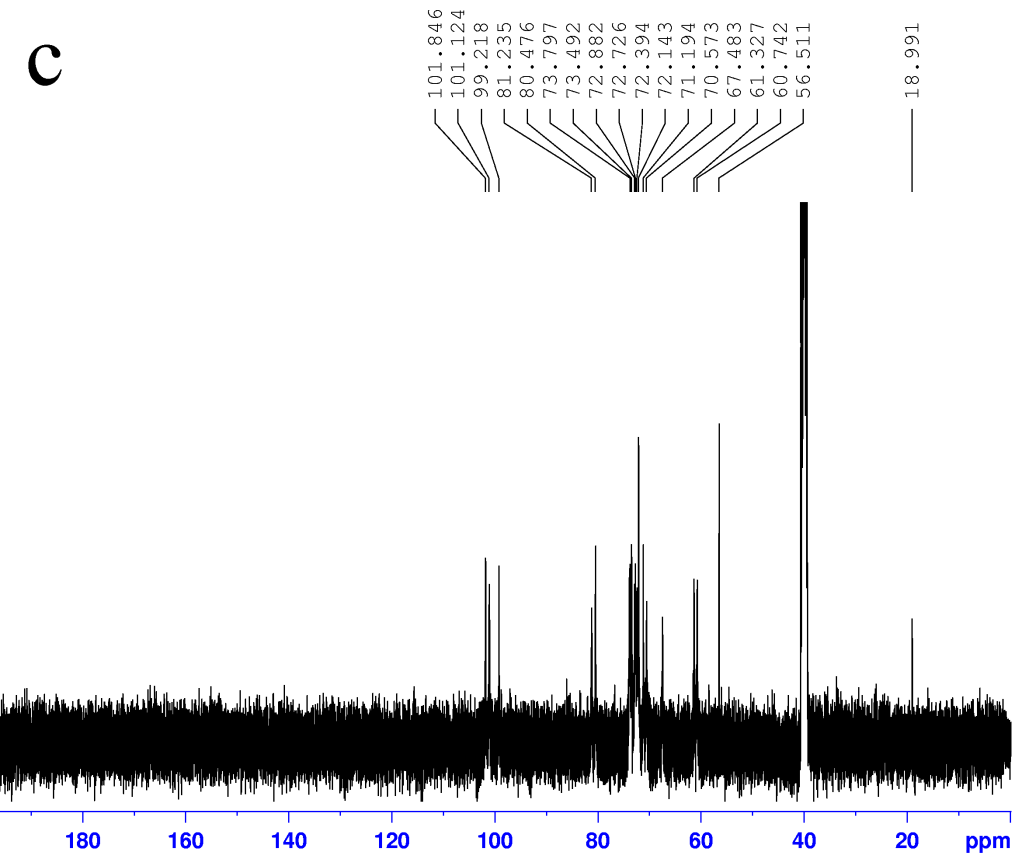

Supplement: Additional file 1: Figure S1. — Structural characterization of the exopolysaccharide with FT-IR spectrum (a), 1H-NMR spectrum (b) and 13C-NMR spectrum (c). (DOCX 275 kb) [file 12896_2017_340_MOESM1_ESM.docx]
